# Policymakers' engagement with ethicists to improve public health in the United States

**DOI:** 10.1016/j.pmedr.2025.103213

**Published:** 2025-08-21

**Authors:** Adam Seth Levine, Andrew G. Shuman

**Affiliations:** a SNF Agora Professor of Health Policy and Management, Johns Hopkins University, 3100 Wyman Park Drive, Baltimore, MD 21211, United States; b Center for Bioethics and Social Sciences in Medicine (CBSSM), University of Michigan Medical School, 1904 Taubman Center, 1500 E Medical Center Dr, Ann Arbor, MI 48105, United States

**Keywords:** Public health, Policymaking, Bioethics, Policy engagement, Partisanship

## Abstract

**Objective:**

To determine how policymakers interact with bioethicists, their interest in future engagement, and their motivation and hesitations to do so.

**Methods:**

Three nationwide surveys of United States policymakers (*N* = 1105) conducted September 15–November 2, 2023, including representative samples of local government elected policymakers (*N* = 459); local managers (*N* = 288); and a diverse (unweighted) sample of state and local civil service workers who provide family support services (*N* = 358). Surveys assessed the prevalence of policymakers' current interactions with bioethicists, unmet desire to engage with them more (and on which policy topics), and hesitations about the value of engaging with them when facing public health challenges.

**Results:**

Only 12.1 % of elected policymakers (95 % CI: 9.0 %,16.3 %), 6.6 % of managers (95 % CI: 4.2 %,10.1 %), and 14.2 % of civil servants (95 % CI: 11.0 %,18.3 %) reported recent interaction with a bioethicist. Yet 40.1 % of elected policymakers (95 % CI: 34.8 %,45.6 %), 40.0 % of managers (95 % CI: 34.1 %,46.2 %), and 47.9 % of civil servants (95 % CI: 42.5 %,53.3 %) expressed an unmet desire for more direct engagement. Partisan differences were present, with Democrats in each sample expressing more unmet desire. Key hesitations to interacting with bioethicists were a perception they would push a political agenda and not share practical information.

**Conclusions:**

Many policymakers wish to seek counsel from those within the bioethics community as they work to promote and protect the health of their community, despite low levels of reported engagement. Amidst widespread calls for more ethically-informed public health policymaking, there is a key opportunity for bioethicists to influence and shape public policy at sub-national levels.

## Highlights

• Direct engagement between bioethicists and policymakers can improve public health.
• We conducted three new, nationwide surveys of local and state policymakers.
• Only 5–15 % of policymakers reported recent interaction with a bioethicist.
• Yet 40–50 % overall want more direct engagement with bioethicists (though partisan differences were present).
• We suggest several ways to connect policymakers and bioethicists.

## 1 Introduction

Public health policy, which often entails balancing risks versus harms, and personal versus population-level rights and obligations, requires an ethics-based lens. Incorporating individual and shared values is essential (Galea, 2024), along with ethically informed guidance regarding how to prioritize and apply them in particular contexts (Leider et al., 2017). Yet as recent and historic public health emergencies have demonstrated, policymakers do not necessarily integrate ethically informed guidance into decision-making when facing policy challenges (Kinder, 2020; Parasidis and Fairchild, 2022; Emanuel and Persad, 2023; Aguilera et al., 2024).

Past experiences provide good reason to believe that deeper engagement at the intersection of bioethics and public health policy would be valuable. At the federal level, this includes five previous Presidential Commissions on Bioethics: executive branch advisory panels charged to “identify and promote policies and practices that ensure scientific research, health care delivery, and technological innovation are conducted in a socially and ethically responsible manner (Presidential Commission).” Prior commissions focused on topics such as how to respond to infectious diseases like Ebola. At the local level, this engagement has informed responses to more specific, community-driven challenges. For example, although aging policy often focuses on medical care, many local policymakers want help thinking about how policy can promote a good life for older adults in their communities more broadly (Berlinger, 2024). Counsel from bioethicists has led policymakers to pose and prioritize the core question: What are the “nature and obligations of good citizenship in aging societies (Berlinger and Solomon, 2018)?” Their counsel has also engendered guides for discussing everyday non-medical goals (e.g., secure housing, food, social connection) to inform local policy decisions.

Examples like these are why bioethicists have argued for a more “ethics-informed” public health policymaking process, and identified several ways in which policymakers can seek counsel from experts in bioethics (Emanuel et al., 2022). In parallel, leaders are expressing growing interest in fostering new connections between bioethics professionals and policymakers (Scully, 2019; Rhodes and Ostertag, 2023). Although bioethics is a relatively young field, it is growing, with over 1700 professionals in the US working in a variety of workplace settings such as academic institutions, bioethics centers, medical centers, and arts and sciences colleges (Pierson et al., 2024). It is also a broad field that includes individuals with diverse and overlapping areas of focus such as medical as well as public health ethics (Callahan and Jennings, 2002).

Thus, while we have reason to suspect that greater interaction would be valuable, and calls by bioethics leaders to increase the *supply* of ethics-based counsel, what we lack is high-quality information on the demand side for a broad range of policymakers. Measuring that – including policymakers' prior experience and desire to interact with bioethicists – is our focus here. We focus on *interacting* with bioethicists, as opposed to only reading their written work, for two reasons. One is the long-standing oral tradition in policymaking, in which policymakers depend more heavily on the spoken rather than the written word (Bogenschneider, 2020). The second is the large research literature on facilitators and barriers of policymakers' use of subject-matter expertise, which finds that collaborative relationships between experts and policymakers (i.e., back-and-forth conversations) are an important facilitator to using this expertise, along with it being relevant, reliable, and timely (and, conversely, having poor access to high-quality, relevant, and timely expertise are key barriers; Oliver et al., 2014; Crowley et al., 2021; Mullin, 2021). While ethical guidance is unlikely to dictate public health policy on its own – political, economic, legal, and scientific information also matter – collaborative relationships between bioethicists and policymakers at least help ensure “robust dialogue that explicitly takes into account ethical considerations (Parasidis and Fairchild, 2022).”

Although attention often focuses on national-level policymakers, here we focus on the local and state levels. In the US, public health governance is shared across levels of government, with substantial authority residing in states and localities. All states have public health departments, with civil servants who are promoting and protecting the health of the community. In addition, there are over 3300 local public health departments, with 77 % serving one or more counties and the remainder serving one or more municipalities or towns. The large majority (81.4 %) are also locally governed, as opposed to solely being units of state government (NACCHO, 2022). The result is that states and localities are both vital locations for policy innovation, and are geographically spread out and thus more accessible to a wider range of bioethicists across the country (Rutkow et al., 2019).

Given these considerations, here we seek to answer the following three research questions about prevalence, unmet desire, and hesitations. First, to what extent do policymakers already engage with bioethicists when facing public health challenges? Second, do they express an unmet desire to engage with them more than they currently are? Third, in what ways might they be hesitant about the value that bioethicists can bring to improving the health of their community?

## 2 Methods

We conducted three nationwide online surveys of policymakers from September 15–November 2, 2023. Two focus at the local level: local government elected policymakers and local government managers. The third includes civil service staff associated with family support services in local and state government. The surveys were fielded by CivicPulse, a nonprofit, nonpartisan organization that maintains large panels of local and state policymakers using publicly-available contact information (updated quarterly) from multiple vendors along with information collected through their own research and correspondence (CivicPulse, 2025).

The overall sample of local government elected policymakers (3825 top elected officials and governing board members) is randomly drawn from a comprehensive list of local elected officials in U.S. township, municipality, and county governments serving communities of 1000 or more. CivicPulse partners with a phone-banking company that calls the office of each these governments listed on the U.S. Census of Governments every three months to acquire the latest contact information. The overall sample of local government managers (1694 city managers and county administrators) is randomly drawn from a comprehensive list of top appointed officials in similar communities. Both were balanced on three key Census-based characteristics associated with the local governments (proportion of college-educated residents, number of residents, and the presidential vote share from the latest election for the county in which the local government is situated).

CivicPulse also builds comprehensive lists of various categories of civil service staff (via keyword searches of GovSpend and their own manual searching). One category is “family support services” and is constructed via searches that include terms related to public health such as mental health, addiction, welfare, youth, and so on (and thus produces a list of people who, broadly speaking, work to protect and promote the public's health). Our overall sample of 3255 civil service staff is randomly drawn from this list.

The total number who completed our survey was 1105, including 459 elected policymakers, 288 managers, and 358 civil servants. It had 26 questions overall, and respondents spent between five and ten minutes on it. The survey was seven screens, and CivicPulse defined “complete” as responding to at least five. Based on the number of completes, our response rates are 12 % for elected policymakers, 17 % for managers, and 11 % for civil servants. These response rates meet and/or exceed those from other nationwide surveys of state and/or local policymakers recently published in several high impact journals (which are between 7 % and 13 %; Pagel et al., 2017; Shaffer et al., 2020; Druckman et al., 2023).

CivicPulse recruited all respondents via email (with one initial email and two follow-ups), and used its standard consent language. Compensation was non-monetary; all respondents received a research brief with the findings several months after fielding (CivicPulse, 2025). Online supplementary material includes question-wording along with details about the construction of survey weights for the elected policymaker and manager samples. This study was approved by the [Johns Hopkins Bloomberg School of Public Health Institutional Review Board], and follows the American Association for Public Opinion Research reporting guidelines (AAPOR, 2025).

### 2.1 Measures

Survey design required being mindful that policymakers may not be familiar with the work or existence of bioethicists. In addition, those within the ethics community frequently describe their work and interdisciplinary expertise in different ways. Given these considerations, the surveys included a brief paragraph to make ethicists' expertise and the inherent diversity of their fields and training understandable to respondents in a way that was broadly accurate and readable. This was vetted with bioethicists representing different professional backgrounds (law, philosophy, and medicine), one local elected policymaker who had previously been a civil servant, and with the CivicPulse leadership. This description read as follows (in which “[decision-makers]” was filled in as appropriate for each sample):

Bioethicists study moral and ethical dilemmas that arise in health-related decisions, including policy. They often interact with *[decision-makers]* as part of their work. They can offer counsel on the issues and value tradeoffs that *[decision-makers]* may confront when working to promote and protect the health of their community. Some *[decision-makers]* are in touch with bioethicists, whereas others are not. And some *[decision-makers]* may not have thought about bioethicists at all prior to this survey.

Notably, this paragraph also employed language that explicitly acknowledged (and legitimized) that some policymakers are in touch with bioethicists and some are not, and some are familiar with them and some are not. This helps establish descriptive norms for the full range of experiences and responses to the questions that follow (Tourangeau et al., 2000).

All questions were otherwise the same for each sample. We asked about whether over the past year policymakers faced in their work and, separately, interacted with bioethicists about four types of decisions regarding the health of their community: how to allocate scarce resources, how to balance individual and government responsibility for community health, how to protect the most vulnerable, and how to navigate inter-governmental decisions that affect community health. We referred to them as “tough decisions” to acknowledge that they may involve hard-to-reconcile value conflicts. We focused on the past year given that questions about retrospective behavior are more reliable when they specify a well-defined time period that is likely to be meaningful to respondents (Tourangeau et al., 2000).

Next, we asked policymakers about their desire to interact with bioethicists in the future when facing these same types of decisions. We report prior engagement in terms of whether respondents had interacted at least once over the past year on at least one decision, and we report unmet desire to engage in terms of whether they wish to interact “somewhat more than now” or “a lot more than now” on at least one decision. In addition, the surveys also included an open-ended question in which policymakers were asked what specific policy issue(s) they would like to engage on.

One of the core challenges with forming new collaborative relationships between policymakers and bioethicists is that they often begin as strangers, and policymakers may be uncertain about relationality – whether bioethicists will relate to them in ways they would like (Levine, 2024). With this in mind, the surveys asked policymakers to identify hesitations they had about interacting with bioethicists, reflecting the information they might share and what the experience of interacting might be like. Lastly, we measured political ideology, partisanship, gender, age, education, and race/ethnicity. All variables are reported for descriptive purposes; we also present unmet desire results by partisanship and gender, given past work finding differences in policymakers' use of research evidence based on these attributes (Hird, 2005).

### 2.2 Statistical analysis

Summary statistics of respondents' demographic characteristics were calculated. To assess prior interaction, we calculated the average percentage of respondents with any recent engagement with bioethicists (along with 95 % confidence intervals). We also calculated prior interaction with other sources of counsel besides bioethicists. To assess unmet desire, we calculated the average percentage who wished to engage with bioethicists more than now (with 95 % confidence intervals). We did so for all respondents, and then by partisanship and gender (and conducted tests of proportions and calculated z-scores to examine differences by these two attributes). We analyzed responses to the open-ended question about what issue(s) they would like to engage on by first creating a coding frame (i.e., a full list of policy issues mentioned across the responses) and then having two coders (both with doctorates in related fields) independently code each response, noting whether the respondent mentioned each issue or not (Cohen's kappa = 0.86; 95 % CI: 0.76,0.96). All discrepancies were resolved via discussion. To understand policymakers' hesitations to interact with bioethicists, we calculated the average percentage of respondents who shared each of eight hesitations (along with 95 % confidence intervals). Throughout the paper, results for elected policymakers and managers use survey weights to increase sample representativeness, unless noted otherwise; weights were unavailable for civil servants. Statistical analyses were conducted using Stata 17 (StataCorp 2021).

## 3 Results

Among elected policymaker respondents, 34.8 % were female, 32.9 % identified as Democrats, and 36.0 % identified as Republicans (unweighted). Among manager respondents, 30.8 % were female, 24.5 % identified as Democrats, and 26.6 % identified as Republicans (unweighted). Among civil servants, 71.0 % were female, 46.1 % identified as Democrats, and 13.9 % identified as Republicans (see supplementary Table S1).

**Fig. 1:**
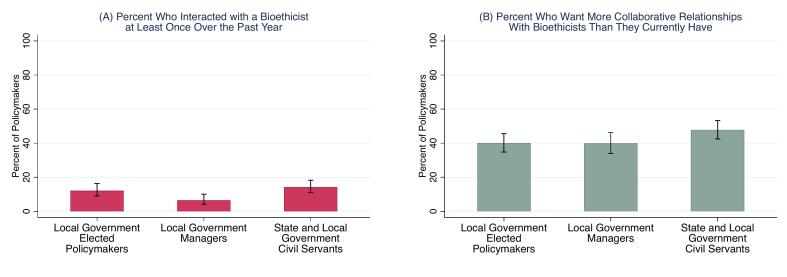
(A) Percent of policymakers in the United States who recently interacted with a bioethicist, and (B) percent who want to engage with bioethicists more than they currently are: September 15–November 2, 2023. Note: (A) reports percent who said that they had interacted with a bioethicist at least once over the past year. (B) reports unmet desire calculated as percent who said that they want to interact with a bioethicist “A lot more than now” or “Somewhat more than now” when facing one or more tough public health decisions. Error bars represent 95 % C.I. Results for elected policymakers and managers are weighted to increase sample representativeness.

Policymakers reported frequently making at least one of the four types of difficult health policy decisions over the past year, including 84.2 % of elected policymakers (95 % CI: 79.8 %,87.8 %), 89.0 % of managers (95 % CI: 84.7 %,92.2 %), and 89.1 % of civil servants (95 % CI: 85.4 %, 92.0 %; see supplementary Table S2 for results by each type of decision). They also reported infrequent interaction with bioethicists. Overall, only 12.1 % of elected policymakers (95 % CI: 9.0 %,16.3 %), 6.6 % of managers (95 % CI: 4.2 %,10.1 %), and 14.2 % of civil servants (95 % CI: 11.0 %,18.3 %) interacted with a bioethicist at least once over the past year (Fig. 1a). This pattern contrasts with other sources of consultation and expertise (Table 1). Across all three types of policymakers, they were far more likely to have interacted with every other source of counsel when facing decisions related to the health of their community.

**Table 1:** Percent of United States policymakers who sought counsel on public health issues from various information sources at least once over the past year: September 15–November 2, 2023.

Potential source of counsel on public health issues	Local government elected policymakers	Local government managers	State and local government civil servants
Other officials in your government	81.7	88.2	49.2
Grassroots/community leaders	69.8	72.9	41.2
Government officials outside your government	66.0	79.5	36.9
Business leaders	60.9	62.0	32.0
Professional association staff	53.0	62.4	42.5
Lobbyists/interest group leaders	26.7	31.7	20.4
Bioethicists	14.5	6.9	16.0

Note: This only includes policymakers who faced a challenging public health decision, which was 84.2 % of elected policymakers, 89.0 % of managers, and 89.1 % of civil servants. This is why the numbers for bioethicists are slightly different than Fig. 1a. Results for elected policymakers and managers are weighted to increase sample representativeness.

Many policymakers want more collaborative relationships with bioethicists than they currently have (Fig. 1b). This includes 40.1 % of elected policymakers (95 % CI: 34.8 %,45.6 %), 40.0 % of managers (95 % CI: 34.1 %,46.2 %), and 47.9 % of civil servants (95 % CI: 42.5 %,53.3 %). Moreover, this unmet desire is higher among those who reported that they faced a public health challenge over the past year, including 46.0 % of elected policymakers (95 % CI: 40.1 %,52.1 %), 41.5 % of managers (95 % CI: 35.2 %,48.1 %), and 52.6 % of civil servants (95 % CI: 46.8 %,58.3 %).

**Fig. 2:**
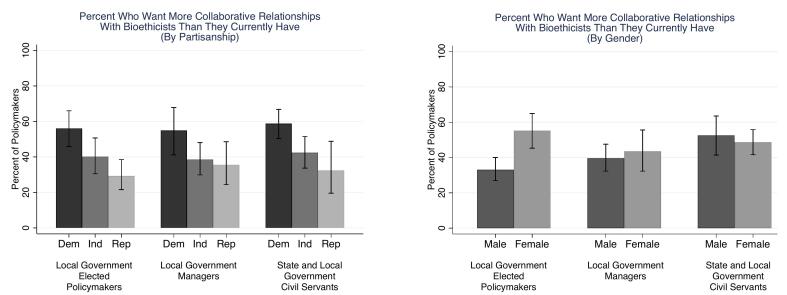
Percent of policymakers in the United States who want to engage with bioethicists more than they currently are, by partisanship and gender: September 15–November 2, 2023. Note: Figure displays percent who said that they want to interact with a bioethicist “A lot more than now” or “Somewhat more than now” when facing one or more tough public health decisions. Error bars represent 95 % C.I. Results for elected policymakers and managers are weighted to increase sample representativeness.

Fig. 2 presents unmet desire stratified by partisanship and gender. Across all three samples, those who identify as Democrats are more likely than independents and Republicans to express an unmet desire for more collaborative relationships with bioethicists. Among elected policymakers, 56.1 % of Democrats expressed unmet desire, as compared with 40.2 % of Independents (*p* < .03; all *p*-values are two-tailed) and 29.3 % of Republicans (*p* < .01). Among managers, 54.9 % of Democrats expressed unmet desire, as compared with 38.6 % of Independents (*p* < .05) and 35.6 % of Republicans (*p* < .04). Among civil servants, 58.8 % of Democrats expressed unmet desire, as compared with 42.4 % of Independents (p < .01) and 32.5 % of Republicans (p < .01).

Gender differences appeared only among elected policymakers, in which 55.3 % of females expressed unmet desire as compared with 33.1 % of males (p < .01). Among managers, 43.6 % of females expressed unmet desire, as compared with 39.7 % of males (*p* = .58), and among civil servants, 48.7 % of females expressed unmet desire, as compared with 52.6 % of males (*p* = .56).

**Fig. 3:**
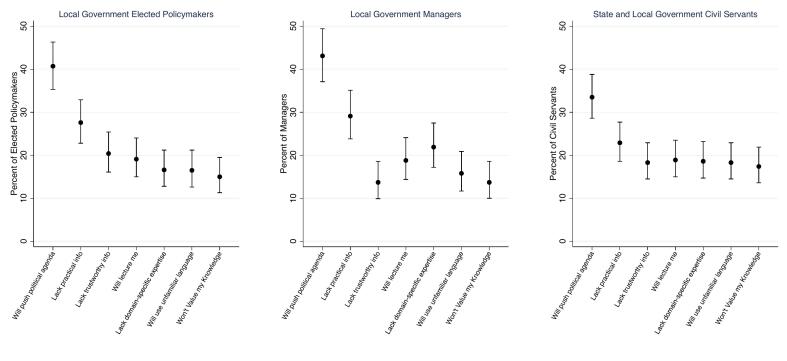
Percent of policymakers in the United States who expressed various concerns about interacting with bioethicists: September 15–November 2, 2023. Note: Figure displays percent who indicated that they shared each hesitation about interacting with a bioethicist. Error bars represent 95 % C.I. Results for elected policymakers and managers are weighted to increase sample representativeness.

On average, policymakers expressed between one and two hesitations about interacting with bioethicists across the three samples. As shown in Fig. 3, no single hesitation was shared by most policymakers. The most common hesitation was identical across the three samples: that bioethicists would push a political agenda. The second-most common hesitation was also shared across all three samples: that bioethicists will lack practical information.

Among those who expressed unmet desire earlier in the survey, 40.6 % of elected policymakers, 36.0 % of managers, and 37.6 % of civil servants also wrote about a particular issue and/or further details about their interest when provided with an open-ended opportunity to do so. Among elected policymakers, the five most common issues were housing/homelessness, health/public health in general, mental health, infectious disease/vaccines, and addiction/substance use disorder. Among managers, the top five were health/public health in general, infectious disease/vaccines, housing/homelessness, water quality, and mental health. Among civil servants, the top five were health/public health in general, infectious disease/vaccines, youth/children well-being, mental health, and housing/homelessness (see supplementary Table S3).

## 4 Discussion

Direct interaction between bioethicists and policymakers is important for a more ethics-informed public health policymaking process. Policymakers routinely seek counsel from several sources such as other officials in their government and leaders from the community when facing challenges on these issues. Thus far, they rarely do so from bioethicists, though many also express dissatisfaction with this status quo. For those with bioethics expertise interested in policy engagement, these results suggest that they will be pushing against an open door.

The next question is how to meet this unmet desire. Among bioethicists interested in policy engagement, our results suggest the value of thinking regionally, not just nationally. One strategy is attending open meetings of the county legislature, municipal board, and related events. Many locales have institutionalized opportunities for public participation (e.g., privilege of the floor), and bioethicists can use that moment to introduce themselves to a broad set of (elected and non-elected) policymakers at once. A second strategy is to work with organizations who may act as formal intermediaries (e.g., universities and professional associations often offer matchmaking services through government relations liaisons; Bednarek et al., 2016). We also suggest consulting published resources such as frameworks on how to develop and evaluate academic-policy engagement interventions (Mäkelä et al., 2024), and case studies of others who have done so (Ehmann et al., 2021). When interacting, given policymakers' concern about receiving practical information, bioethicists must ensure they are responsive to policymakers' specific needs and legal context (Brownson et al., 2006). Given policymakers' concern about a political agenda, bioethicists can (if applicable) emphasize their prior interactions across party lines and be explicit about how their counsel reflects values that transcend partisanship (Feinberg and Willer, 2019).

Partisan differences in survey responses are noteworthy and echo previous work showing how partisanship influences public health (for example, Covid-19 vaccination rates between Republicans and Democrats, as well as infection and mortality rates in majority-Republican versus majority-Democrat counties, significantly differed; Shin and McCarthy, 2013; Kannan and Veazie, 2018; Motta, 2024; Pacheco et al., 2024). To be sure, partisanship is not always visible to outsiders, as not all local elections are partisan, and the party affiliations of civil servants and appointed officials are rarely publicly available. Yet our results suggest that bioethicists' guidance is likely to be viewed through a partisan lens (see Fig. 3). The need for bioethicists to “honor the value of opposition” and embrace a non-partisan and non-adversarial approach to engagement is critical (Chambers, 2023).

Our data have limitations. For instance, it is possible that expressions of unmet desire may not be genuine or engender a change in future behavior (i.e., they may be influenced by social desirability). We anticipated this by using survey design techniques that minimize the potential impact of social desirability. When asking a0b.out policymakers' interactions with bioethicists we employed descriptive norm language to explicitly acknowledge (and legitimize) that some may want to engage and some may not. We also used an open-ended question (What specific policy issue(s) would you like to speak about?) that required more effort to respond to, relative to the closed-ended unmet desire questions. In addition, elsewhere policymakers do not seem shy about sharing non-socially desirable opinions. For instance, only 3–9 % of respondents across the three surveys reported being very or extremely familiar with bioethicists at the beginning of the survey.

Still, we do not interpret our finding that 40–50 % of policymakers express unmet desire as indicating that all of them would respond affirmatively to outreach at any single moment. Limited capacity and various agenda priorities make that unlikely. That is why outreach methods (e.g. attending in-person county-wide meetings) are potentially valuable because they can reach several policymakers at once.

One other limitation relates to selection bias. It is difficult to achieve high response rates in surveys of active policymakers (though our response rates meet or exceed other recently-published policymaker surveys). Survey weights help account for selection bias, and we used them for the elected policymakers and managers. Survey weights were not available for the civil servant sample and thus self-selection is a concern when interpreting these findings.

## 5 Conclusion

Many local and state policymakers who are responsible for promoting and protecting the health of their community do not seek counsel from bioethicists when facing policy challenges in their work. Our data indicate that, contrary to this current state, many policymakers express an unmet desire for such counsel (though partisan differences are present). Our findings suggest a key opportunity for bioethicists to influence and shape public policy.

## CRediT authorship contribution statement

**Adam Seth Levine:** Writing – review & editing, Writing – original draft, Visualization, Validation, Software, Resources, Project administration, Methodology, Investigation, Funding acquisition, Formal analysis, Data curation, Conceptualization. **Andrew G. Shuman:** Writing – review & editing, Writing – original draft, Conceptualization.

## Declaration of competing interest

This research was supported by a grant to Levine by the Greenwall Foundation. The Greenwall Foundation did not affect the design and submission of this research.

## Data Availability

Data will be made available on request.

## Appendix A Supplementary data

Supplementary material Image 1
