# Supplementary material for: Policymakers' engagement with ethicists to improve public health in the United States
Source: Prev Med Rep. 2025 Aug 21;58:103213. doi: 10.1016/j.pmedr.2025.103213 (PMC12418881; doi:10.1016/j.pmedr.2025.103213)
Supplement: Supplementary file 1 — Supplementary material [file mmc1.pdf]

ONLINE SUPPLEMENTARY INFORMATION
"Policymakers' engagement with ethicists to improve public health in the United States"
By Adam Seth Levine and Andrew Shuman
***Preventive Medicine Reports*, 2025.**

Survey Text

Making tough decisions

--Over the past year, how often have you faced tough decisions about how to protect the health of the most vulnerable in your community?

More than once a week
Once a week
Once or twice a month
Once or twice over the entire year
Never

--Resources are essential for ensuring the health of your community, such as improving youth mental health, staffing mobile crisis units, responding to infectious disease outbreaks, and many other pressing issues. But often there's not enough to meet the need.
Over the past year, how often have you faced tough decisions that entailed deciding how to allocate scarce money and/or staff to ensure the health of your community?

More than once a week
Once a week
Once or twice a month
Once or twice over the entire year
Never

--Protecting and promoting the health of the community can entail tough decisions in which you have to decide how much individuals should be held responsible for their own choices and how much the government should be involved.
Over the past year, how often have you faced decisions that you felt entailed this kind of value conflict?

More than once a week
Once a week
Once or twice a month
Once or twice over the entire year
Never

--Sometimes decisions made at other parts or levels of government might compromise the health of people in your community. These may relate to a variety of topics such as providing for an aging population, medical care for gender transition, vaccination, abortion etc.
From your perspective, how often does this happen to your community?

More than once a week
Once a week
Once or twice a month

Once or twice over the entire year
Never

<u>Prior experiences</u>

Over the past year, when you've faced tough decisions related to the health of your community, how often have you sought counsel from each of the following?

- Other officials in your own government
- Other officials outside your own government
- Local business leaders
- Local grassroots/community leaders
- Lobbyists/Interest group leaders
- Staff at national or state-level municipality/town/county/state organizations (e.g. NACo, NATaT)

More than once a week
Once a week
Once or twice a month
Once or twice over the entire year
Never

[Before asking about prior interactions/desired future interactions with bioethicists, respondents received this paragraph, which aimed to make ethicists' expertise understandable to respondents in a way that was broadly accurate and readable (and was vetted with bioethicists representing professional backgrounds in law, philosophy, and medicine). In addition, it used language that legitimized (via the use of descriptive norm language) the idea that policymakers may or may not have interacted with bioethicists or be familiar with them at all.]

Bioethicists study moral and ethical dilemmas that arise in health-related decisions, including policy. They often interact with *[decision-makers]* as part of their work. They can offer counsel on the issues and value tradeoffs that *[decision-makers]* may confront when working to promote and protect the health of their community. Some *[decision-makers]* are in touch with bioethicists, whereas others are not. And some *[decision-makers]* may not have thought about bioethicists at all prior to this survey.

[The [decision-makers] part was filled in differently for each survey.]

--Over the past year, on average how often have you interacted with bioethicists to talk about policy issues?

More than once a week
Once a week
Once or twice a month
Once or twice over the entire year
Never

--Prior to taking this survey, how familiar or unfamiliar were you with what bioethicists do?

Extremely familiar
Very familiar
Somewhat familiar
Neither familiar nor unfamiliar
Somewhat unfamiliar
Very unfamiliar
Extremely unfamiliar

[Note: Responses to this question largely validated an ex ante concern expressed above about policymakers' pre-existing lack of familiarity with bioethicists. Only 8.1% of electeds, 3.9% of managers, and 8.9% of civil servants reported that they were either very or extremely familiar with what bioethicists do.]

Unmet desire

[As with the introduction to the previous section of the survey, here we again used language ("whether you might wish") to explicitly legitimize the fact that respondents may or may not wish to engage with bioethicists.]

Looking to the future, you may face tough decisions when working to promote and protect the health of your community. These next few questions ask about whether you might wish to seek counsel from bioethicists when faced with decisions like these.

--Looking ahead, how much would you like to be in touch with bioethicists to talk through tough decisions about how to allocate scarce money and/or staff time toward the goal of ensuring the health of your community?

A lot more than now
Somewhat more than now
The same as now
Somewhat less than now
A lot less than now

--Looking ahead, how much would you like to be in touch with bioethicists to talk through tough decisions in which you have to decide how much individuals should be held responsible for their own health choices and how much the government should be involved in order to ensure the health of the overall community?

A lot more than now
Somewhat more than now
The same as now
Somewhat less than now
A lot less than now

--Looking ahead, how much would you like to be in touch with bioethicists to talk through tough decisions about how to protect the health of the most vulnerable in your community?

A lot more than now
Somewhat more than now

The same as now
Somewhat less than now
A lot less than now

--As mentioned earlier, sometimes decisions made at other parts or levels of government might compromise the health of people in your community. Looking ahead, how much would you like to be in touch with bioethicists to talk through how to navigate these situations?

A lot more than now
Somewhat more than now
The same as now
Somewhat less than now
A lot less than now

Open-ended policy question

--What specific policy issue(s), if any, would you be most interested in speaking with bioethicists about?

Hesitations

When you think about interacting with bioethicists, which of the following challenges (if any) do you expect may occur? *Please check all that apply.*

They may not have domain-specific expertise
They may not have trustworthy information
They may not have practical information
They may not value my knowledge and experience
They may lecture me
They may use unfamiliar language
They may push a political agenda

Demographic questions (these are standard question-wordings included at the end of all CivicPulse surveys)

In general do you think of yourself as:
Very conservative
Somewhat conservative
Moderate, middle of the road
Somewhat liberal
Very liberal
Not sure

[Note: The finding that concern about pushing a political agenda was the top-rated hesitation is perhaps not surprising, as recent surveys of bioethicists in the United States find that 87% identify as liberal (Pierson et al., 2024) as compared with only 19.2% of elected policymakers, 21.6% of managers, and 32.9% of civil servants in our surveys.]

Generally speaking, do you usually think of yourself as a:
Democrat
Republican
Independent
Other party

What is your gender?
Man
Woman
Prefer to self-describe

When were you born?
[Drop down menu included 19 binned years, starting with “1920 or earlier” through “2006 or later”]

What is the highest level of education you have completed?
Less than high school
High school graduate
Technical/trade school
Some college
College graduate
Some graduate school
Graduate degree

Which of the following best describes your race/ethnicity? Please check all that apply.
Asian/Pacific Islander
Black/African American
Hispanic/Latinx
Native American
Middle Eastern
Mixed Race
White
Prefer to self-describe

Further Information on Data Collection and Sample Representativeness

Table S1 includes characteristics of respondents in each sample. Within each attribute, numbers may not add up to total number of respondents due to respondents answering “other” and/or question non-response.

Table S1:
Characteristics of United States policymakers in each sample (unweighted data): September 15-November 2, 2023

**Attribute**	**Local government elected policymakers, No. (%) (N=459)**	**Local government managers, No. (%) (N=288)**	**State and local government civil servants, No. (%) (N=358)**
Government type			
County	83 (18.1)	30 (10.4)	NA
Municipality	263 (57.3)	217 (75.4)	NA
Township	113 (24.6)	41 (14.2)	NA
Local	NA	NA	175 (48.9)
State	NA	NA	183 (51.1)
Sex			
Female	127 (34.8)	73 (30.8)	191 (71.0)
Male	238 (65.2)	164 (69.2)	78 (29.0)
Partisanship			
Democrat	129 (32.9)	57 (24.5)	136 (46.1)
Independent	122 (31.1)	114 (48.9)	118 (40.0)
Republican	141 (36.0)	62 (26.6)	41 (13.9)
Race and ethnicity			
Black, Asian, Hispanic, Native American, Middle Eastern, or Mixed Race[a]	47 (13.0)	23 (9.7)	144 (51.4)
White	316 (87.1)	214 (90.3)	136 (48.6)
Education			
College degree	277 (69.6)	217 (87.5)	227 (76.2)
No college degree	121 (30.4)	31 (12.5)	71 (23.8)
Age			
Born 1920-1965	244 (68.5)	110 (46.6)	51 (19.0)
Born 1966-1980	86 (24.2)	92 (39.0)	122 (45.4)
Born 1981-present	26 (7.3)	34 (14.4)	96 (35.6)

[a]*These were each separate response categories in the surveys, yet the survey firm grouped them to prevent identifiability.*

Table S2 displays the percentage of policymakers who faced each of the following decisions at least once over the past year.

Table S2:
Percent of United States policymakers who faced the following decisions at least once over the past year: September 15-November 2, 2023

**Type of decision**	**Local government elected policymakers (N=459)**	**Local government managers (N=288)**	**State and local government civil servants (N=358)**
How to protect the most vulnerable	73.4%	79.5%	78.8%
How to allocate scarce resources	65.5%	66.2%	57.8%
How to trade-off individual responsibility versus government	59.1%	69.8%	70.9%
How to respond to other levels of government	70.1%	70.7%	80.7%
Faced at least one of the above decisions	*84.2%*	*89.0%*	*89.1%*

Table S3 displays the five most common issues that respondents from each sample mentioned in response to the open-ended question. Here the denominator is those who expressed unmet desire to engage with bioethicists and mentioned an issue in response to the open-ended question. As noted in the main text, we first created a coding frame (i.e., a full list of all policy issues mentioned across all of the responses). Then, two coders (both with doctorates in related fields) independently coded each response, noting whether the respondent mentioned each issue (coded as zero or one). The coding scheme included 31 possible issues (plus one "Other" category), underscoring the way in which local political agendas vary quite a bit. Given the open-ended structure of this question, respondents were free to mention as many issues as they would like. Each respondent mentioned on average only 1.36 issues, and in general the overwhelming majority of codes were quickly and easily assessed as zero's.

Table S3:
Five most common issues United States policymakers would like to discuss with bioethicists: September 15-November 2, 2023

**Local government elected policymakers**	**Local government managers**	**State and local government civil servants**
Housing/homelessness (25.9%)	Health/public health in general (19.3%)	Health/public health in general (17.0%)
Health/public health in general (24.5%)	Infectious disease/vaccines (16.8%)	Infectious disease/vaccines (11.9%)
Mental health (12.5%)	Housing/homelessness (15.9%)	Youth/children well-being (11.9%)
Infectious disease/vaccines (10.9%)	Water quality (14.3%)	Mental health (10.2%)
Addiction/substance use disorder (10.8%)	Mental health (13.7%)	Housing/homelessness (8.5%)

The randomly-sampled contact lists (for electeds and managers) were balanced on three key Census-based characteristics associated with the local governments (proportion of college-educated residents, number of residents, and the presidential vote share from the latest election for the county in which the local government is situated).

Tables S4a and S4b were used by CivicPulse to calculate survey weights for the local elected policymakers and local government managers datasets. The tables compare the overall sampling frames with the samples collected for these studies.

All results reported in the main text use survey weights based on these numbers, provided by CivicPulse and calculated using a post-stratification raking procedure using the three variables below.

Table S4a:
Sample representativeness for local elected policymakers: September 15-November 2, 2023

Type	Population Size Median	College Education Median	Republican Vote Share Median
Sampling Frame	5,020	0.22	0.59
Sample	8,230	0.27	0.51

Table S4b:
Sample representativeness for local government managers: September 15-November 2, 2023

Type	Population Size Median	College Education Median	Republican Vote Share Median
Sampling Frame	7,740	0.27	0.52
Sample	7,570	0.26	0.52
